# Multi-disciplinary team for early gastric cancer diagnosis improves the detection rate of early gastric cancer

**DOI:** 10.1186/s12876-017-0711-9

**Published:** 2017-12-06

**Authors:** Lianjun Di, Huichao Wu, Rong Zhu, Youfeng Li, Xinglong Wu, Rui Xie, Hongping Li, Haibo Wang, Hua Zhang, Hong Xiao, Hui Chen, Hong Zhen, Kui Zhao, Xuefeng Yang, Ming Xie, Bigung Tuo

**Affiliations:** 1 Department of Gastroenterology, Affiliated Hospital, Zunyi Medical College, Zunyi, 563003 China; 2 Digestive Endoscopy Center, Affiliated Hospital, Zunyi Medical Colleage, Zunyi, China; 3 Department of Pathology, Affiliated Hospital, Zunyi Medical College, Zunyi, China; 4 Department of Anesthesiology, Affiliated Hospital, Zunyi Medical College, Zunyi, China; 5 Department of Gastrointestinal Surgery, Affiliated Hospital, Zunyi Medical College, Zunyi, China

**Keywords:** Early gastric cancer, Diagnosis, Intensive gastroscopy, Multi-disciplinary team

## Abstract

**Background:**

Gastric cancer is a frequent malignant tumor worldwide and its early detection is crucial for curing the disease and enhancing patients’ survival rate. This study aimed to assess whether the multi-disciplinary team (MDT) can improve the detection rate of early gastric cancer (EGC).

**Methods:**

The detection rate of EGC at the Digestive Endoscopy Center, Affiliated Hospital, Zunyi Medical College, China between September 2013 and September 2015 was analyzed. MDT for the diagnosis of EGC in the hospital was established in September 2014. The study was divided into 2 time periods: September 1, 2013 to August 31, 2014 (period 1) and September 1, 2014 to September 1, 2015 (period 2).

**Results:**

A total of 60,800 patients’ gastroscopies were performed during the two years. 61 of these patients (0.1%) were diagnosed as EGC, accounting for 16.44% (61/371) of total patients with gastric cancer. The EGC detection rate before MDT (period 1) was 0.05% (16/29403), accounting for 9.09% (16/176) of total patients with gastric cancer during this period. In comparison, the EGC detection rate during MDT (period 2) was 0.15% (45/31397), accounting for 23% (45/195) of total patients with gastric cancer during this period (*P* < 0.05). Univariate and multivariate logistic analyses showed that intensive gastroscopy for high risk patients of gastric cancer enhanced the detection rate of EGC in cooperation with Department of Pathology (OR = 10.1, 95% CI 2.39–43.3, *P* < 0.05).

**Conclusion:**

MDT could improve the endoscopic detection rate of EGC.

**Electronic supplementary material:**

The online version of this article (10.1186/s12876-017-0711-9) contains supplementary material, which is available to authorized users.

## Background

Gastric cancer is the fourth frequent malignant tumor and the second leading cause of cancer related death in the world. Every year about 738,000 people die of gastric cancer, and the overall 5-year survival rate is about 20% [1, 2]. The prevalence of gastric cancer has district and gender differences. The incidence of gastric cancer in the North American women is lowest, with average incidence of 3.4 /100000 people, whereas it is highest in Asian men, with average incidence of 26.9/100000, especially in Japan, South Korea, and China [3–9]. Although the medical advances have reduced gastric cancer mortality, the gastric cancer remains the second leading cause of cancer-related death in Asia [10]. Early gastric cancer (EGC) was first defined by the Japan Gastroscopy Association as an adenocarcinoma limited to the mucosa and submucosa, regardless of lymph node metastasis [11]. Based on this standard, the 5-year survival rate of patients with EGC after surgical treatment has reached 90%, whereas 5-year survival rate of advanced gastric cancer is still less than 30% [12, 13]. Therefore, the early detection of gastric cancer is crucial to enhance survival rate of patients. Japan is the best country in screening EGC work [14]. The National Cancer Center of Japan reported that the ratio of EGC patients in all gastric cancer patients increased from 22% in the 1960s to 75% in the 2000s [15]. China is a high risk area of gastric cancer, with about 400,000 added gastric cancer patients and about 350,000 patients died of this malignant disease each year, and the new and dead patients account for 40% of patients with gastric cancer in the world [16], while the detection rate of EGC in China accounts only for 5% to 20% of total gastric cancer. The low detection rate of EGC in China may be not only related to endoscopist’s awareness, experience, ability to identify EGC, and but also related to lack of coordination and cooperation between different departments in the Hospital including Gastroenterology, Pathology, Gastrointestinal Surgery, and Endoscopy Center.

The diagnosis and treatment of gastric cancer should be completed by multi-disciplinary team (MDT) according to Clinical Practice Guidelines of National Comprehensive Cancer Network (NCCN) for gastric cancer in the 2013 version [17]. Improving the detection rate of EGC not only relies on the ability of endoscopist, but also needs multidisciplinary cooperation, especially the cooperation of endoscopist and pathologist. To date, few studies have assessed the association between MDT and the detection rate of EGC. Therefore, in this study, we investigated whether MDT could improve the detection rate of EGC.

## Methods

### Basic information about the endoscopy center and endoscopists

The Digestive Endoscopy Center of Affiliated Hospital of Zunyi Medical College is one of the largest endoscopy centers in China that meets international standards. Approximately 30,000 gastroscopies were performed annually during the past three years. The endoscopists in the Center are all skilled in endoscopic diagnosis and treatment, and each endoscopist has an experience performing over 3000 gastroscopic examinations.

### MDT methods to improve the detection of EGC

MDT for diagnosis and treatment of EGC was established in September 2014, which contains the Departments of Digestive Endoscopy Center, Gastroenterology, Gastrointestinal Surgery, Anesthesiology, and Pathology. The discussion meeting of EGC MDT was held once a month. Digestive Endoscopy Center, Department of Gastroenterology and Department of Pathology were the main members of MDT meeting. The measures of MDT were as follows. First, endoscopists were trained through lectures, watching photos and videos, on-site teaching, and participating discussion meeting for EGC patients in the Center once a week to enhance their awareness and ability to identify EGC. Two senior endoscopists (HuihaoWu and Lianjun Di) made intensive gastroscopies for high-risk patients of gastric cancer (ie, those with atrophic gastritis, gastric ulcer, stomach surgery history, and first-degree relatives of gastric carcinoma patients). During this process, painless and comfortable gastroscopy was made to facilitate careful examination; mucus decomposing, antifoaming and spasmolytic agents were used to improve the visibility of the gastric mucosa; and standardized gastroscopy photography, white light endoscopy (WLE) indigo carmine staining, narrow band imaging (NBI), and magnifying endoscopy were performed to improve the detection of EGC. Secondly, the Center strengthened the cooperation with Department of Gastroenterology. The gastroenterologists in outpatient service screened high-risk patients of gastric cancer, and then the endoscopist made intensive gastroscopies for the high risk patients. It is most important to use a magnifying endoscopy for intensive gastroscopy. Magnifying endoscopy with narrow-band imaging (M-NBI) can make suspicious lesions more visible. The magnifying endoscopic diagnosis of EGC was determined according to the vessel plus surface (VS) classification system, including an irregular microvascular and/or microsurface pattern together with a clear demarcation line [18]. Thirdly, a regular communication and discussion for the diagnosis of EGC patients between endoscopist and pathologist was made once a week. Fourthly, strengthening cooperation with Department of Gastrointestinal Surgery, all patients with gastric carcinoma who were not suitable for endoscopic submucosal dissection (ESD) were discussed multidisciplinarily within one week to determine the scope and grade of the lesion and operation way. For the patient diagnosed as gastric cancer for many times by endoscopist, but repeated biopsies did not support the diagnosis of gastric cancer, multidisciplinary discussion made a decision whether it needs further surgery. Finally, strengthening cooperation with Department of Anesthesiology, painless endoscopy can eliminate the patient’s fear, avoid nausea and vomiting reaction, and slow gastric peristalsis, which is contributive to further intensive gastroscopy for suspicious lesions.

### Study design

All gastroscopies performed from September 1, 2013 to September 1, 2015 were reviewed. The study was divided into 2 periods, period 1 (September 1, 2013 to August 31, 2014) and period 2 (September 1, 2014 to September 1, 2015) according to the time of MDT establishment. The endoscopists to undergo endoscopy were same during the two periods. Pathological diagnosis for EGC was performed by gastrointestinal pathologists according to the revised Vienna classification [19]. Mucosal high-grade neoplasia (including high-grade adenoma/dysplasia), noninvasive carcinoma (carcinoma in situ), suspicious for invasive carcinoma, and intramucosal carcinoma were diagnosed as EGC. The diagnosis of EGC before MTD (period 1) was determined by endoscopist according to endoscopic and histological examinations, without collaboration and communication of MDT. The EGC detection rates before and during MDT were compared. The factors affecting the detection of EGC were analyzed by two endoscopists (HC.W. and LJ.D.). This study was approved by the ethics committee of Zunyi Mecial College, and all patients provided written informed consent for the procedures before endoscopy.

Demographic and clinical characteristics of all patients were evaluated, including age, gender, status (outpatient/inpatient), gastrointestinal symptoms such as abdominal pain and vomiting, past medical history (mainly atrophic gastritis, gastric ulcer), and whether first-degree relatives of gastric carcinoma patients. Endoscopic characteristics to be assessed in the patients with EGC included the site and general morphology of the lesion, surface microstructure, and vascular characteristics.

### Statistical analysis

Statistical analysis was processed by using the SPSS PC statistic package. The age with mean ± standard deviation was evaluated using independent samples t test. The Pearson Chi-Square (*χ* ^*2*^) test was applied for the detection rates of EGC between different cooperation departments, sex ratio, and ratio of inpatient and outpatient before and during MDT. The factors affecting the detection of EGC were assessed by univariate and multivariate logistic analyses. Odds ratio (OR) and 95% confidence intervals (CIs) were determined for significant variables found on multivariate analysis. *P* < 0.05 was considered statistically significant.

## Results

### Rates of EGC detection

From September 1, 2013 to September 1, 2015, the gastroscopies of a total of 60,800 patients were performed in the Digestive Endoscopy Center of Affiliated Hospital of Zunyi Medical College. Among 60,800 patients, 61 patients (0.1%) were diagnosed as EGC, accounting for 16.44% of total 371 diagnosed gastric cancer patients during this period. The EGC detection rate by endoscopists before MDT was 0.05%, accounting for 9.09% of the all diagnosed gastric cancer patients during the period 1. In contrast, the EGC detection rate during MDT was 0.15%, accounting for 23% of the all diagnosed gastric cancer patients during the period 2 (Table 1).

**Table 1:** The comparison of EGC detection rate before and during MDT

	Before MDT	During MDT	All	*P* value	*X* ^*2*^
NG	29,043	31,397	60,800	–	–
EGC/NG(%)	16/29403 (0.05%)	45/31397 (0.15%)	61/60800 (0.1%)	<0.001	11.975
EGC/GC (%)	16/176 (9.09%)	45/195 (23%)	61/371 (16.44%)	<0.001	19.593

*NG* number of gastroscopies, *EGC* number of early gastric carcinoma, *GC* number of total gastric carcinoma

### Characteristics of EGC

**Fig. 1:**
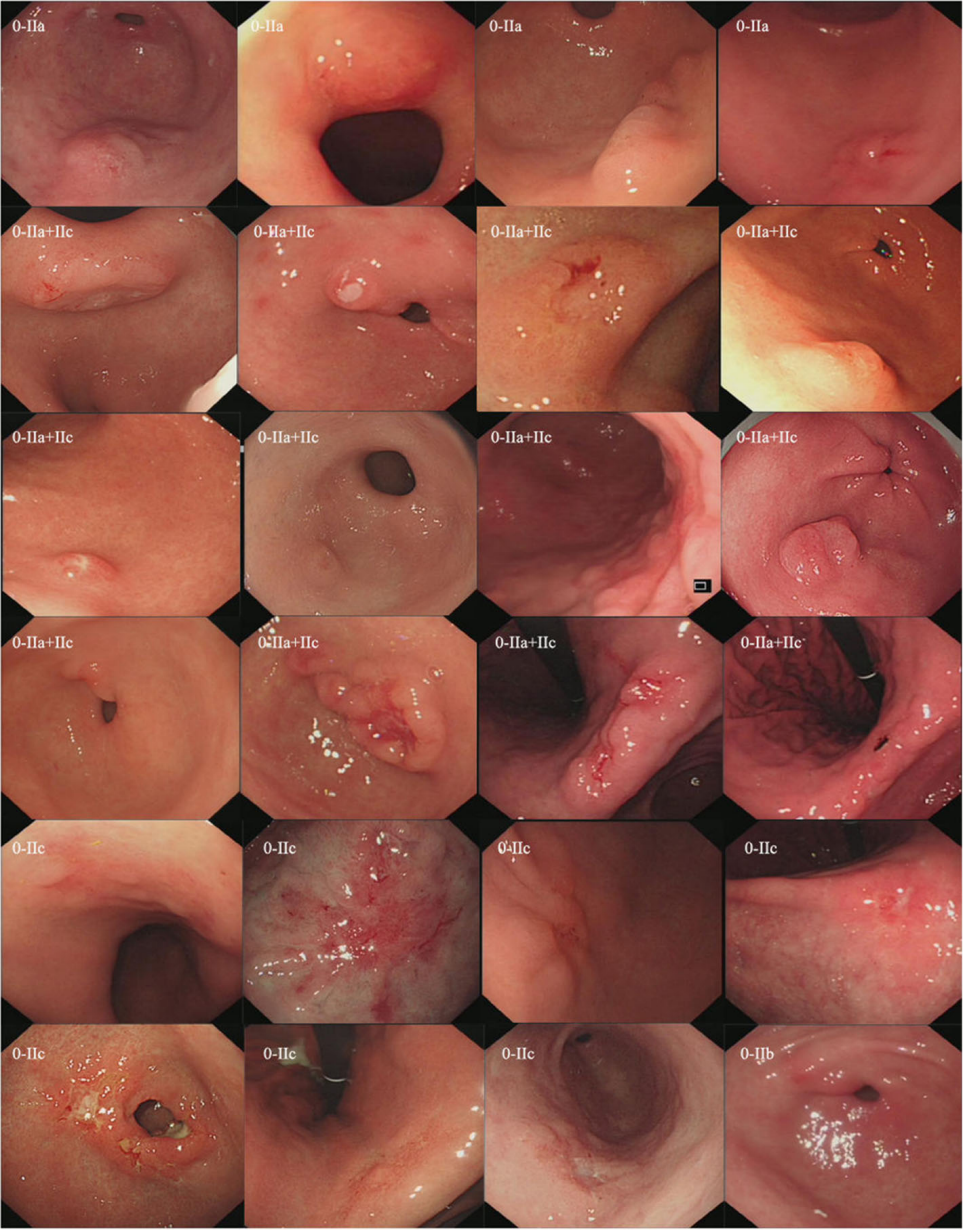
Representative EGC lesion images under white light imaging

As shown in Table 2, among the 61 EGCs, 4 were located at the gastric fundus, 3 at the lesser curvature of the gastric corpus, 7 at the greater curvature of the gastric corpus, 4 at the posterior of the gastric corpus, 1 at the anterior of the gastric corpus, 13 at the gastric angle, and 29 at the gastric antrum. 1 was protruding type (0-I), 10 were surface protruding type (0–IIa), 15 were surface depressed type (0–IIc), 2 were flat type (0–IIb), 20 were mixed type (0–IIa + IIc), 2 were mixed type (0–IIc + IIa), and 11 were depressed type (0–III). In general, EGC has various morphological characteristics and the lesion is very subtle. Among the 61 EGCs, images of 24 cases are shown in Fig. 1, including the general morphology of the lesion under the white light imaging (WLI) for these EGCs.

**Table 2:** Sites and the general morphologic and histologic characteristics of EGCs

	Before MDT	During MDT
Lesion location		
Gastric fundus	0	4
Lesser curvature of gastric corpus	2	1
Greater curvature of gastric corpus	5	2
Posterior of gastric corpus	0	4
Anterior of gastric corpus	0	1
Gastric angle	2	11
Gastric antrum	7	22
Morphological characteristic		
0–I	0	1
0–IIb	0	2
0–IIa	2	8
0–IIc	4	11
0–IIa + IIc	6	14
0-IIc + IIa	0	2
0-III	4	7
Histological characteristic		
Total number of HGIN	9	25
Tub.1and Tub.2.	4	12
Por 1	3	6
Sig	0	2
Depth of tumor invasion		
T1a	13	38
T1b	3	7

Tub.1, well-differentiated adenocarcinoma;Tub.2, moderately-differentiated adenocarcinoma; Por 1, poorly-differentiated adenocarcinoma; Sig, signet-ring cell carcinoma; EGC, early gastric cancer; HGIN**,** high-grade intraepithelial neoplasias. T1a, Tumor confined to the mucosa (M); T1b, Tumor confined to the submucosa (SM)

### Examples of EGC

**Fig. 2:**
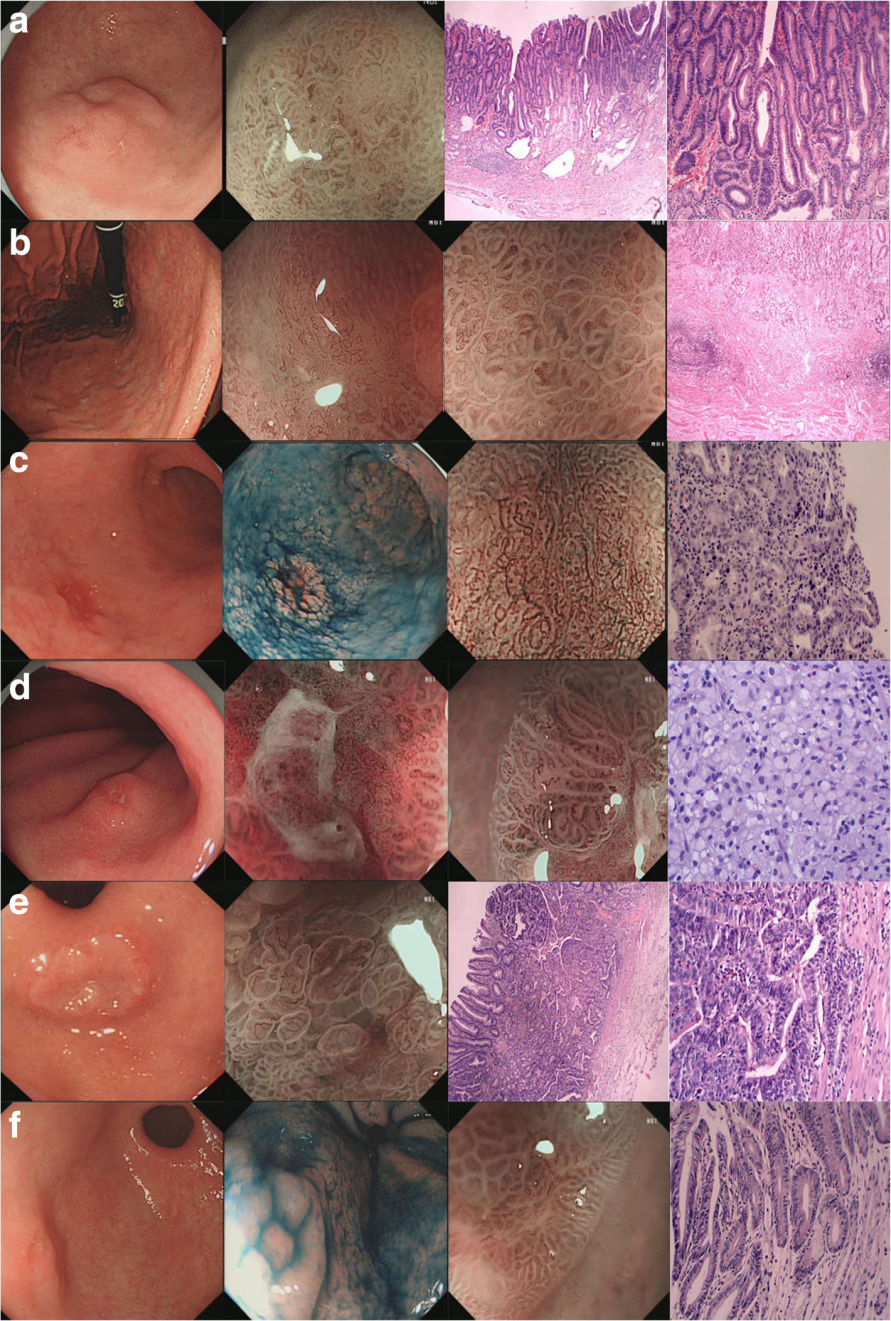
Typical EGC lesions detected after we underwent intensive endoscopy for high-risk patient of gastric carcinoma. **a** Endoscopic image for 0-IIa in white light imaging and magnifying endoscopy and histopathological image. **b** Endoscopic image for 0-IIb in white light imaging and magnifying endoscopy and histopathological image. **c** Endoscopic image for 0-IIc in white light imaging and indigo carmine staining and magnifying endoscopy and histopathological image. **d** Endoscopic image for 0-IIa + IIc in white light imaging and magnifying endoscopy and histopathological image. **e** Endoscopic image for 0-IIa + IIc in white light imaging and magnifying endoscopy and histopathological image. **f** Endoscopic image for 0-IIa + c in white light imaging and indigo carmine staining and magnifying endoscopy and histopathological image

Figure 2 shows endoscopic and histopathologic images of 6 typical EGCs, classified as 0–IIa (A), 0–IIb (B), 0–IIc (C), and 0–IIa + IIc (D, E, F) lesions, respectively. Representative endoscopic and histopathologic images of other patients with EGC are shown in Additional file 1 and Additional file 2. Among the 6 EGCs, 4 were located at the gastric antrum, 2 were located at the gastric corpus and all had typical appearance on M-NBI, including an irregular microvascular and an irregular microsurface pattern with a demarcation line. Pathological examinations of ESD- or surgery-resected specimens showed that there was high-grade intraepithelial neoplasia, signet ring cell carcinoma, or moderately- differentiated adenocarcinoma in these patients.

### Impact of MDT on the detection rate of EGC

**Fig. 3:**
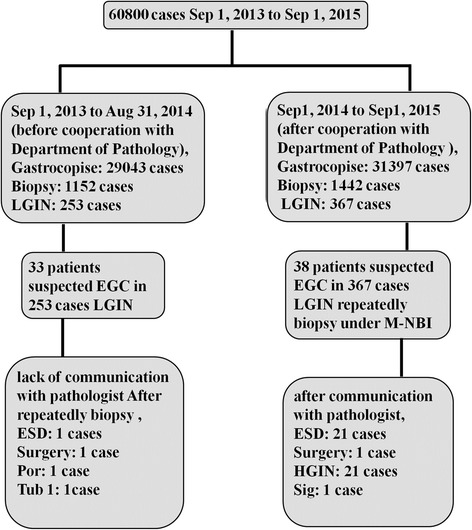
Flow chart of the detection of EGC before and after cooperation with Department of Pathology. HGIN**,** high-grade intraepithelial neoplasia; LGIN, low-grade intraepithelial neoplasia; Por 1, poorly differentiated adenocarcinoma; Sig, signet-ring cell carcinoma; Tub.1, well-differentiated adenocarcinoma

During September 2013 to September 2015, 60,800 gastroscopies were performed at our center, 2594 patients were underwent biopsies and 620 patients were diagnosed as low-grade intraepithelial neoplasia. Before MDT, from September 1, 2013 to August 31, 2014, 29,043 gastroscopies were performed, 1152 patients were underwent biopsies, and 253 patients were diagnosed as low-grade intraepithelial neoplasia. 33 patients were suspected EGC under white light imaging (WLI) among 253 patients. After repeatedly biopsies, only 2 patients were diagnosed as EGC. Pathological examination of ESD- or surgery resected specimens showed that 1 was well-differentiated adenocarcinoma and 1 was poorly-differentiated adenocarcinoma. During MDT, from September 1, 2014 to September 1, 2015, 31,397 gastroscopies were performed, 1442 patients were underwent biopsies, and 397 patients were initially diagnosed low-grade intraepithelial neoplasia. 38 patients were suspected EGC under WLI among the 397 patients, and after repeatedly targeted biopsies under M-NBI, 21 patients were diagnosed as high-grade intraepithelial neoplasia and 1 patient was diagnosed as signet ring cell carcinoma among the 38 patients by cooperative consultation with Department of Pathology. Finally, 21 patients with high-grade intraepithelial neoplasia were resected with ESD and 1 patient of signet ring cell carcinoma was treated by surgery. The detection rate of EGC in the low-grade intraepithelial neoplasia was markedly increased from 0.7% to 5.9% before and after cooperation with Department of Pathology (Fig. 3).

Before cooperation with Department of Gastrointestinal Surgery, tissue biopsies were obtained repeatedly from 11 patients with typical characteristics of the advanced gastric cancer under WLI, and pathological examination showed that all were low-grade intraepithelial neoplasia. Only 2 patients were treated and confirmed as EGC with advanced gastric cancer appearance. After cooperation with Department of Gastrointestinal Surgery, there are 10 patients with typical characteristics of the advanced gastric cancer under WLI, but pathological examination showed low-grade intraepithelial neoplasia. 6 patients were treated and confirmed as EGC with advanced gastric cancer appearance by consultation with Department of Gastrointestinal Surgery. The detection rate of EGC in the low-grade intraepithelial neoplasia was markedly increased from 0.7% to 1.6% before and after cooperation with Department of Gastrointestinal Surgery.

**Fig. 4:**
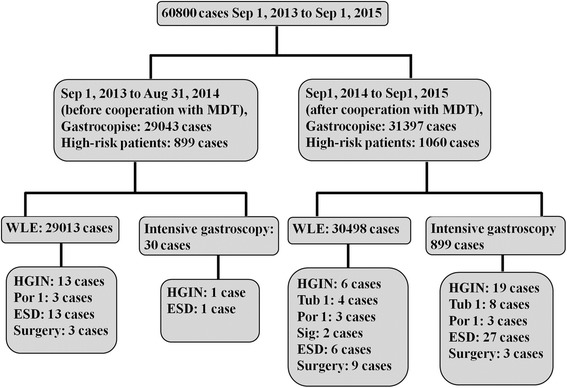
Flow chart of the detection of EGC on intensive gastroscopy for high risk patients of gastric cancer before and after MDT. ESD, endoscopic submucosal dissection; HGI, high-grade intraepithelial neoplasia; Por.1, poorly differentiated adenocarcinoma; Sig, signet-ring cell carcinoma; Tub.1, well-differentiated adenocarcinoma; WLE, white light endoscopy

After high-risk patients of gastric cancer were screened by the gastroenterologists at outpatient service, endoscopists further made intensive gastroscopy for the high risk patients. The detection rate of EGC in high-risk patients by intensive gastroscopy was 3.3%, whereas the detection rate of EGC only by white light endoscopy was 0.5% (Fig. 4). Further results by univariate and multivariate logistic analyses showed that the cooperation with Department of Pathology (OR = 10.1, 95% CI 2.39–43.3, *P* < 0.05) and intensive gastroscopy for high-risk patients (OR = 28.3, 95% CI 19.6–40.7, *P* < 0.001) were independently associated with the detection of EGC. Intensive gastroscopy for high risk patients of gastric cancer enhanced the detection rate of EGC in cooperation with Department of Pathology. Moreover, 899 of 31,397 (2.8%) gastroscopies were performed under intensive gastroscopy during MDT, compared with 30 of 29,013 (0.1%) gastroscopies before MDT. There were no significant differences in gender and age of patients, tissue biopsy rate, the number of high-risk patients, and the number of painless gastroscopy before and during MDT (Table 3).

**Table 3:** Univariate and multivariate logistic analyses of related influencing factors on the detection of EGC

	Univariate Analysis			Multivariate analysis	
	Before MDT	During MDT	*P* value	OR	*P* value
Gender (male)	13,069/29043	14,599/31397	**>**0.05	**—**	**—**
Age, mean(SD)	43(8)	45(9)	**>**0.05	**—**	**—**
TBR	1152/29403	1442/31397	<0.001	**—**	**—**
High-risk patients	899/ 29,403	1060/31397	**>**0.05	**—**	**—**
CWDA	18,564/29403	19,665/31397	<0.05	**—**	**—**
CWDP	2/ 29,043	22/31397	<0.001	10.1 (2.39–43.3)	<0.05
CWDGS	2/29043	6/31397	**>**0.05	**—**	**—**
MDT	16/29043	45/31397	<0.001	2.60 (1.47–4.60)	<0.001
IG	30/29043	893/31397	<0.001	28.3 (19.6–40.7)	<0.001

Gender, number of male in total patients; TBR, number of patients with tissue biopsy in total patients; High-risk patients, number of high-risk patients in total patients; CWDA, cooperation with Department of Anesthsiology, number of painless gastroscopy in total gastroscopy; CWDP, cooperation with Department of Pathology, number of diagnosed patients with EGC in total patients; CWDGS, cooperation with Department of Gastroenterology surgery, number of diagnosed patients with EGC in total patients; MDT, number of diagnosed patients with EGC in total patients; IG, intensive gastroscopy for high-risk patients of gastric cancer, number of diagnosed patients with EGC in total patients

## Discussion

The current study demonstrates that MDT for EGC diagnosis plays an important role in improving the detection rate of EGC. Enhancement of EGC detection rate needs not only the endoscopist’s improvement of the ability to detect EGC, but also close cooperation and regular consultation with multidisplinary.

Gastroscopic diagnosis of EGC is difficult, because lesion of EGC is very complex or subtle so that it may be missed during gastroscopy. In addition, endoscopist’s less attention and poor recognition ability for EGC, non-standardized biopsy, and lack of communication and cooperation between departments are also related to the missed diagnosis and misdiagnosis of EGC. Therefore, training for endoscopist, standardized endoscopic examination, and cooperation and communication of MDT should be done to enhance the detection rate of EGC.

Since MDT for EGC was established, we have been keeping on improving endoscopist’s ability to detect EGC by regular consultation with multidisplinary, especially in cooperation with Department of Pathology. Endoscopist’s awareness for EGC is strengthened and tissue biopsy rate and positive rate are increased. Meanwhile, pathologists also enhance their diagnostic level for EGC. During MTD (period 2), among 22 patients who were initially misdiagnosed as low-grade intraepithelial neoplasia, 21 were diagnosed as high-grade intraepithelial neoplasia and 1 was diagnosed as signet ring cell carcinoma after discussion with Department of Pathology. We think that the cause to result in initial pathological misdiagnosis may be related to the accuracy of pathological diagnosis, the judgment of tissue differentiation degree, pathologist’s recognition ability for EGC, lack of standardized specimen processing and slice, and different diagnostic criteria. There are differences in pathological diagnosis between pathologists because of the lack of uniform diagnostic criteria, especially in the diagnosis for high-grade intraepithelial neoplasia and well-differentiated adenocarcinoma. Well-differentiated adenocarcinoma is misdiagnosed as high-grade intraepithelial neoplasia and severe dysplasia is misdiagnosed as moderate dysplasia. A study showed that 16% patients diagnosed as adenocarcinoma by Japanese pathologists were ascribed to dysplasia by the pathologists in western countries, whereas, in western countries, 90% patients diagnosed as dysplasia by pathologists were ascribed to gastric cancer by Japanese scholars [20]. For this situation, our center strengthens the cooperation with Department of Pathology and unifies diagnostic criteria for EGC. The detection rate of EGC is obviously improved.

High-risk patients of gastric cancer are recognized to have high risk suffering from gastric cancer. We made intensive gastroscopy on high-risk patients screened by gastroenterologists. The result showed that the detection rate of EGC in the high-risk patients by intensive gastroscopy was 3.3%, whereas the detection rate of EGC only by white light endoscopy was 0.5%, demonstrating that the intensive gastroscopy for the high risk patients could enhance the detection of EGC. The previous study also showed that targeted biopsy under M-NBI on doing intensive gastroscopy could improve the detection rate of EGC [21]. We think it is feasible and recommendable measure to make further intensive gastroscopy for high-risk patient of gastric cancer in China, because there are a large number of population and poor economic condition in China and it is impractical to make intensive gastroscopy for each patient.

Clinically, there are some patients with typical signs of advanced gastric cancer under WLE, but repeated biopsies show low-grade intraepithelial neoplasia. In this study, our result showed that the detection rate of EGC in the patients with endoscopic signs of gastric cancer, but pathological result of low-grade intraepithelial neoplasia, was enhanced from 0.7% to 1.6% after the cooperation with Department of Gastrointestinal Surgery. In addition, although univariate and multivariate analyses showed no statistical significance in the number of painless gastroscopy before and during MDT, we think that the painless and comfortable gastroscopy is important to detect EGC in cooperation with Department of Anesthesiology. The painless gastroscopy could make gastric peristalsis slow, without nausea and vomiting response, and eliminate the patient’s fear, which is contributive to further intensive gastroscopy for suspicious lesions.

In general, our study showed that MDT could enhance the detection rate of EGC. Although it is a single-center study and a summary of the experience of a single endoscopy center, the study includes a large sample and it is applicable to other endoscopy centers, especially to those in which the detection rate of EGC by endoscopist is not satisfactory.

## Conclusions

MDT for EGC can improve the detection rate of EGC by endoscopist. Intensive gastroscopy for high-risk patients of gastric cancer and cooperation with Department of Pathology contribute to the detection of EGC.

## Additional files

Additional file 1: Data of patients with early gastric cancer before MDT. The data contain representative endoscopic and histopathologic images of 16 patients diagnosed as early gastric cancer before MDT. (PDF 1057 kb)

Additional file 2: Data of patients with early gastric cancer during MDT. The data contain representative endoscopic and histopathologic images of additional 39 patients diagnosed as early gastric cancer during MDT. (PDF 2710 kb)

## Abbreviations

CI: confidence interval
EGC: early gastric cancer
ESD: endoscopic submucosal dissection
MDT: multi-disciplinary team
M-NBI: magnifying endoscopy with narrow-band imaging
OR: odds ratio
WLE: white light endoscopy
WLI: white light imaging
