# Supplementary material for: Multi-disciplinary team for early gastric cancer diagnosis improves the detection rate of early gastric cancer
Source: BMC Gastroenterol. 2017 Dec 6;17:147. doi: 10.1186/s12876-017-0711-9 (PMC5719518; doi:10.1186/s12876-017-0711-9)
Supplement: Supplementary file 1 — Data of patients with early gastric cancer before MDT. The data contain representative endoscopic and histopathologic images of 16 patients diagnosed as early gastric cancer before MDT. (PDF 1057 kb) [file 12876_2017_711_MOESM1_ESM.pdf]

The data are representative endoscopic and histopathologic images of 16 patients diagnosed as early gastric cancer before MDT.

Case 1, female, 62y, 0-IIa+IIc, HGIN

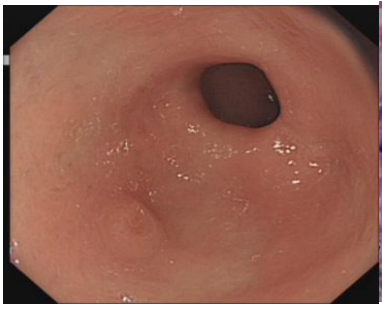
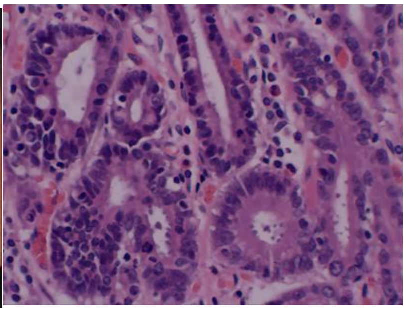

Case 2, female, 49y, 0-IIc, Por 1

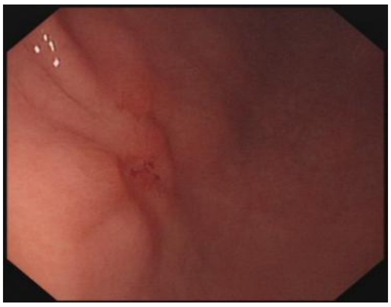
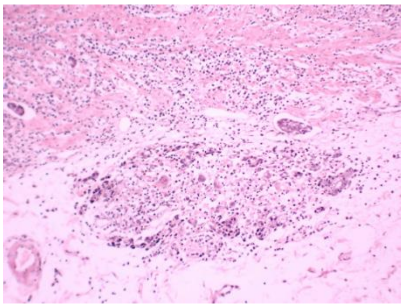

Case 3, male, 62y, 0-IIc, Por 1

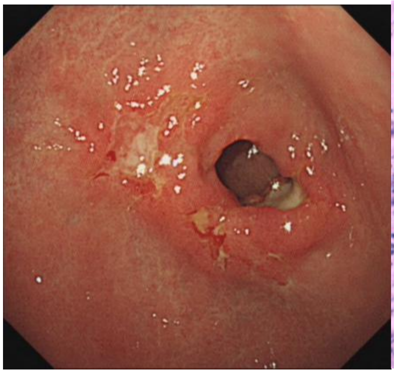
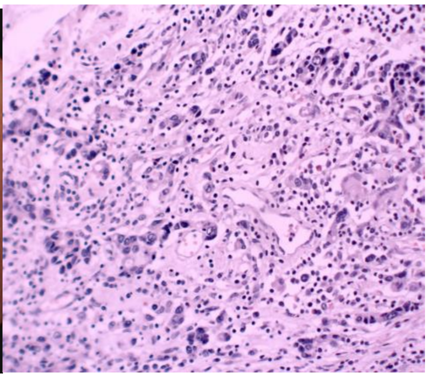

Case 4, female, 71y, 0-IIc, HGIN

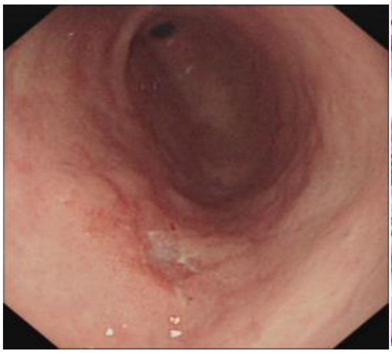
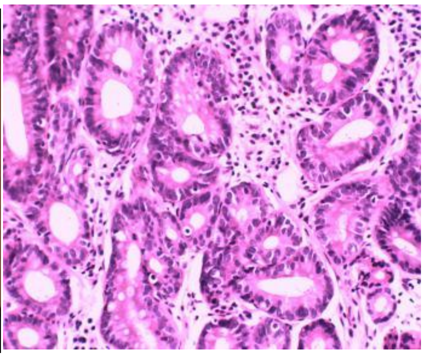

Case 5, male, 53y, 0-IIa+IIc, HGIN, Tub 1、2

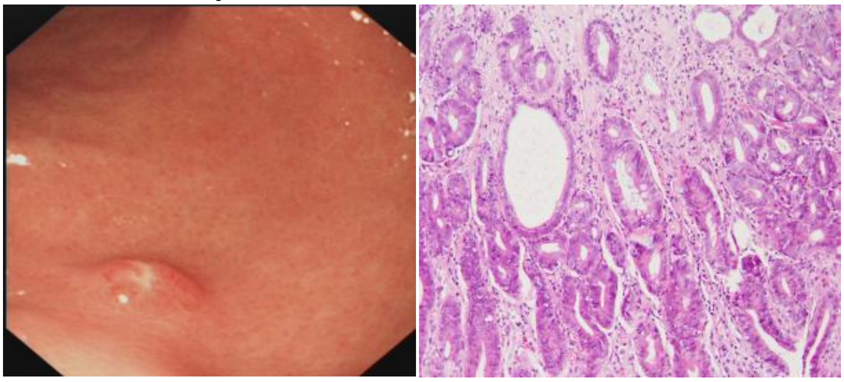

Case 6, female, 54y, 0-IIa, HGIN

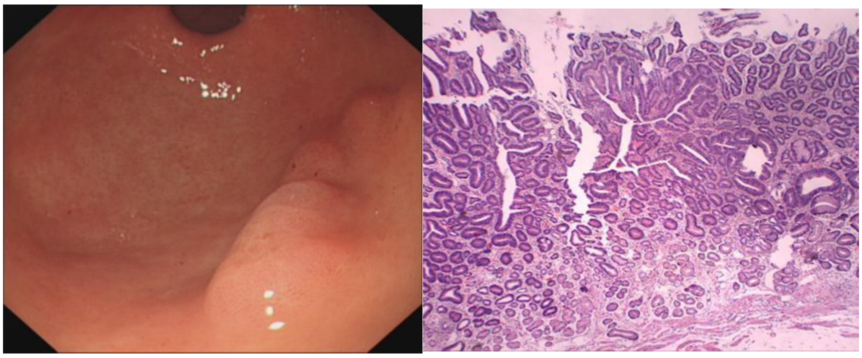

Case 7, male, 71y, 0-IIa, HGIN

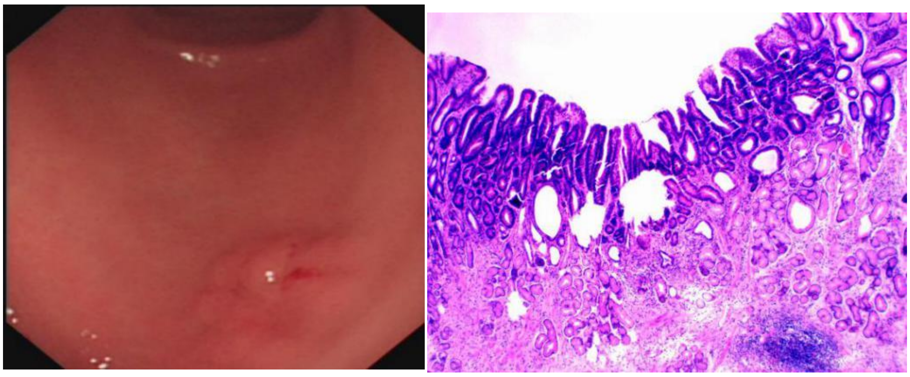

Case 8, male, 67y, 0-IIa+IIc, HGIN

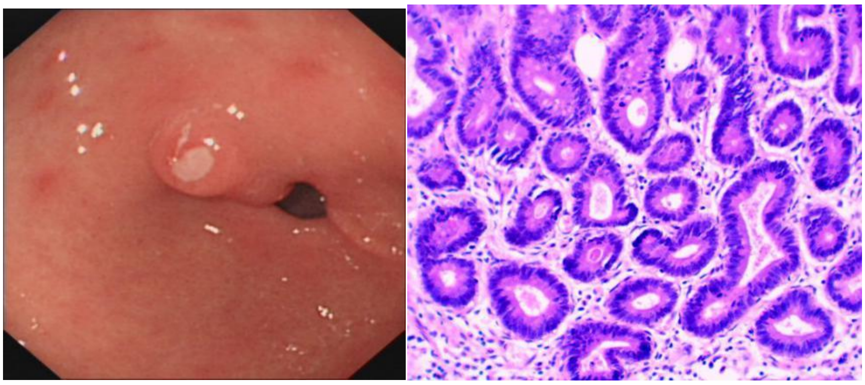

Case 9, male, 51y, 0-IIa+IIc, HGIN

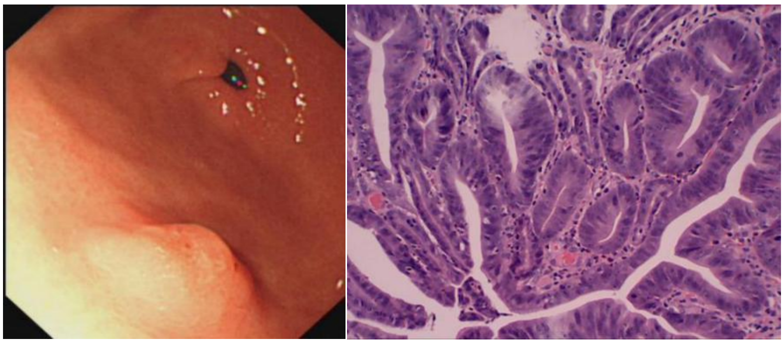

Case 10, female, 63y, 0-IIa+IIc, HGIN

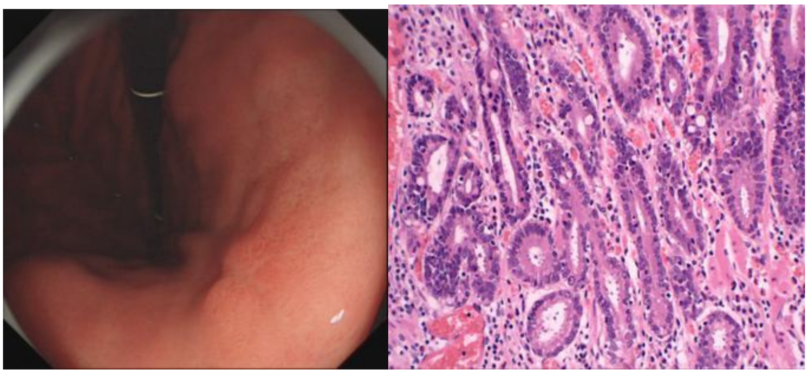

Case 11, female, 72y, 0-IIc, HGIN

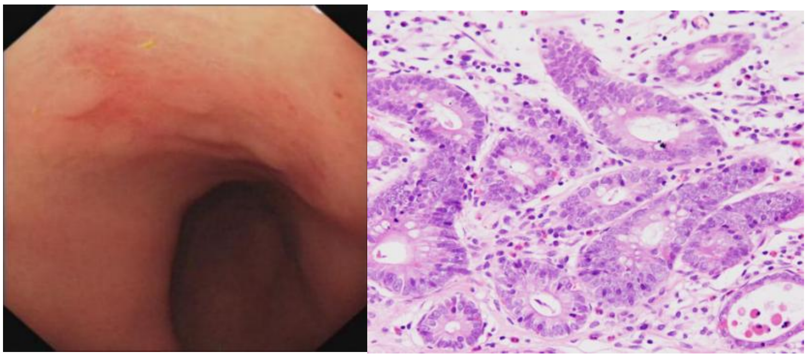

Case 12, male, 71y, 0-IIa+IIc, HGIN

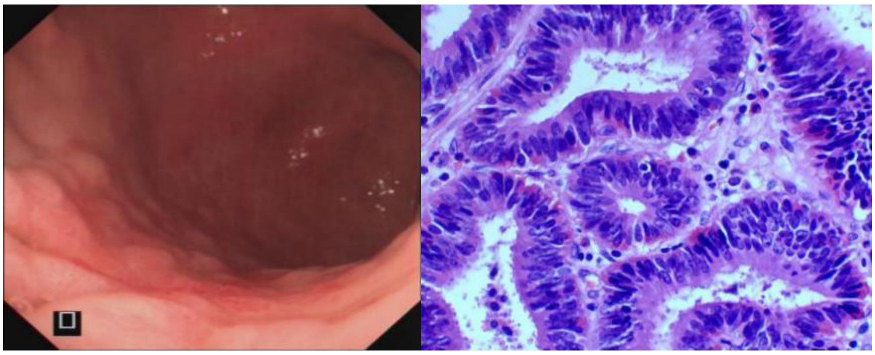

Case 13, female, 68y, 0-III, Tub1, 2

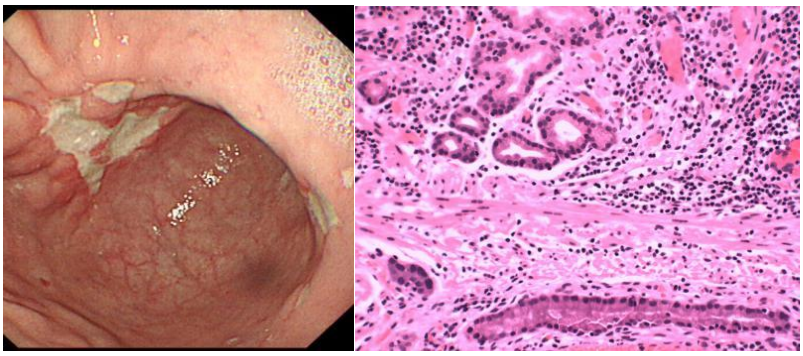

Case 14, male, 44y, 0-III ,Tub1, 2

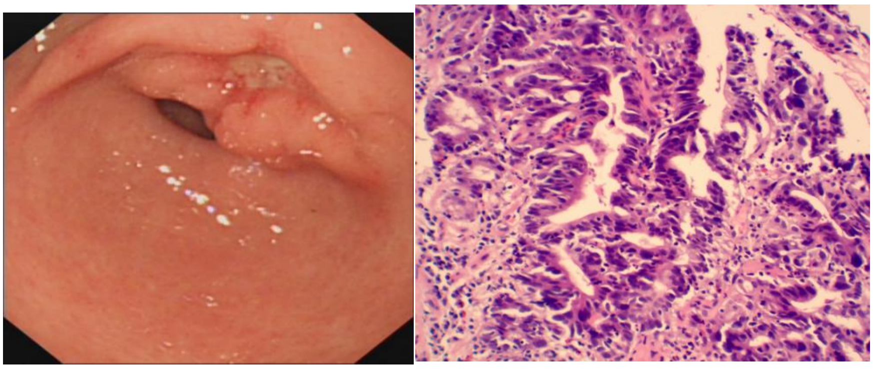

Case 15,female,66y, 0-III, Tub1, 2

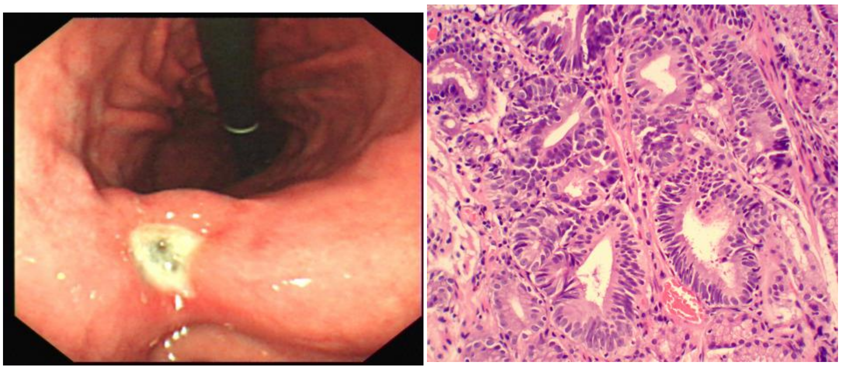

Case 16,male,59y, 0-III   Por 1

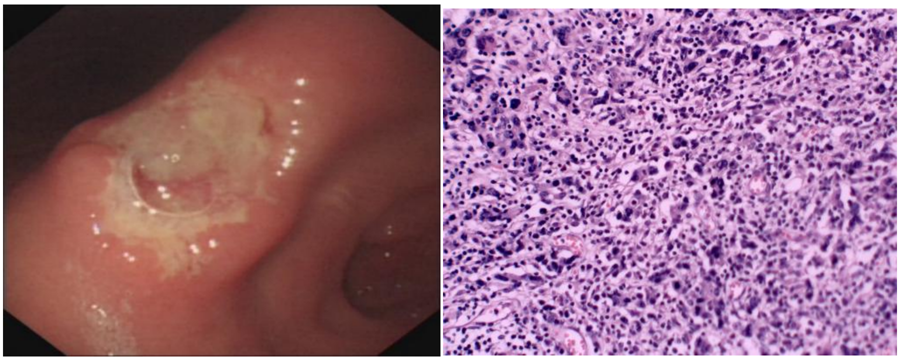

Notes: EGC, early gastric cancer; HGIN**,** high-grade intraepithelial neoplasias; MDT, multi-disciplinary team; Por 1, poorly-differentiated adenocarcinoma; Sig, signet-ring cell carcinoma; Tub1, well-differentiated adenocarcinoma; Tub 2, moderately-differentiated adenocarcinoma.
