# Supplementary material for: Multi-disciplinary team for early gastric cancer diagnosis improves the detection rate of early gastric cancer
Source: BMC Gastroenterol. 2017 Dec 6;17:147. doi: 10.1186/s12876-017-0711-9 (PMC5719518; doi:10.1186/s12876-017-0711-9)
Supplement: Supplementary file 2 — Data of patients with early gastric cancer during MDT. The data contain representative endoscopic and histopathologic images of additional 39 patients diagnosed as early gastric cancer during MDT. (PDF 2710 kb) [file 12876_2017_711_MOESM2_ESM.pdf]

The data are representative endoscopic and histopathologic images of additional 39 patients diagnosed as early gastric cancer during MDT.

Case 1, male, 58y, 0- IIc, Por 1

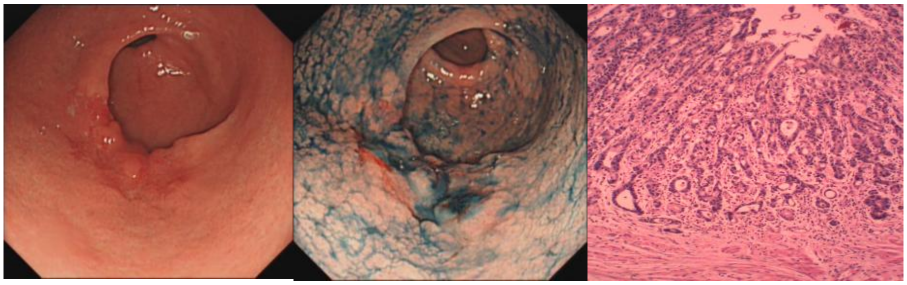

Case 2, male, 52y, 0-III, Por 1

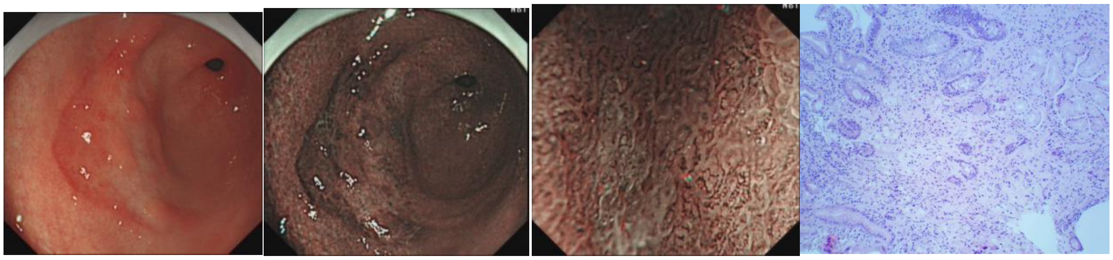

Case 3, female, 62y, 0-IIc,   Por 1

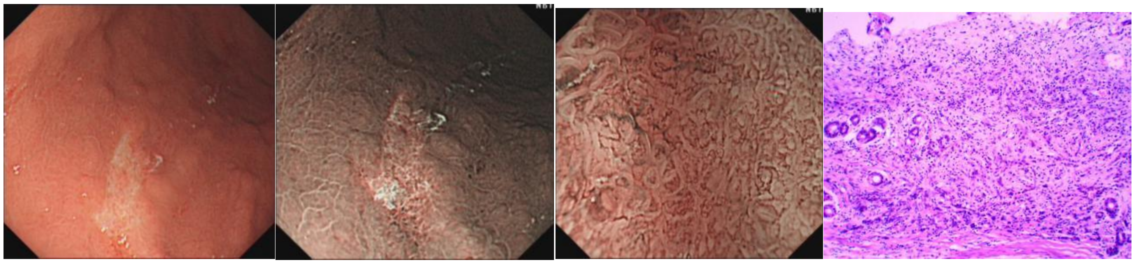

Case 4, male, 48y, 0-IIa+IIc, Por 1

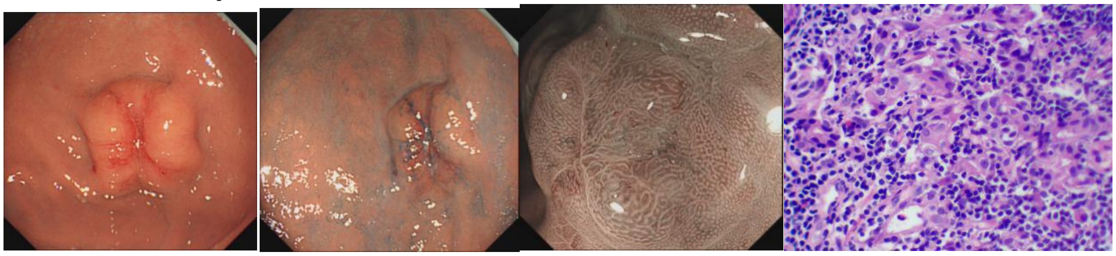

Case 5, female, 46y, 0-III, Por 1

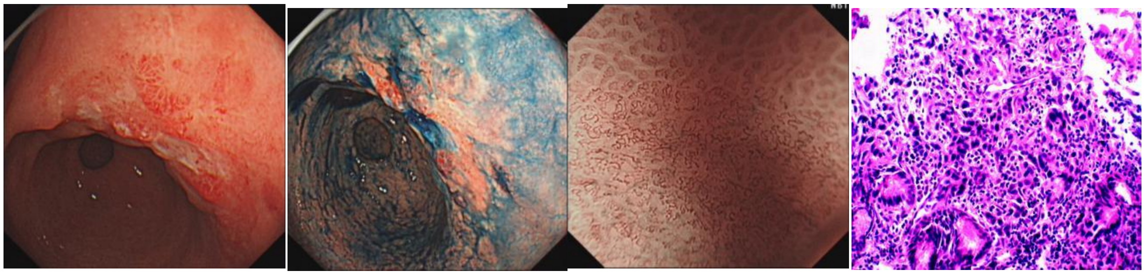

Case 6, male, 80y, 0-III, Por 1

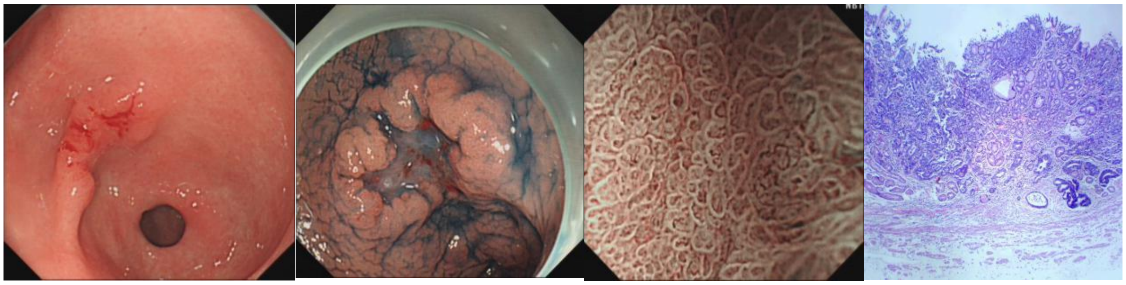

Case 7, male, 72y, 0-IIc+IIa, HGIN

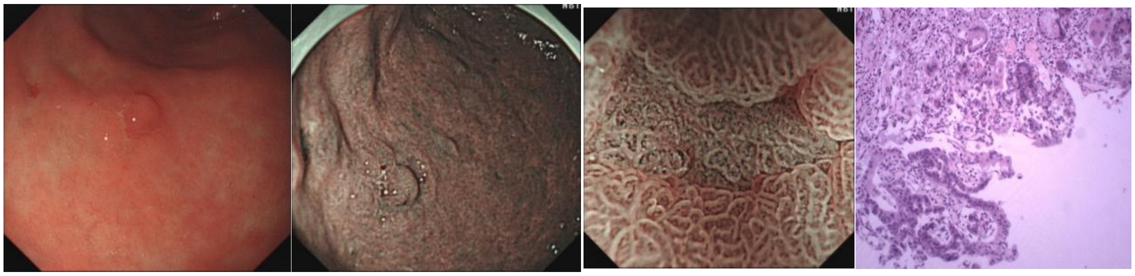

Case 8, male, 69y, 0-I, Tub1

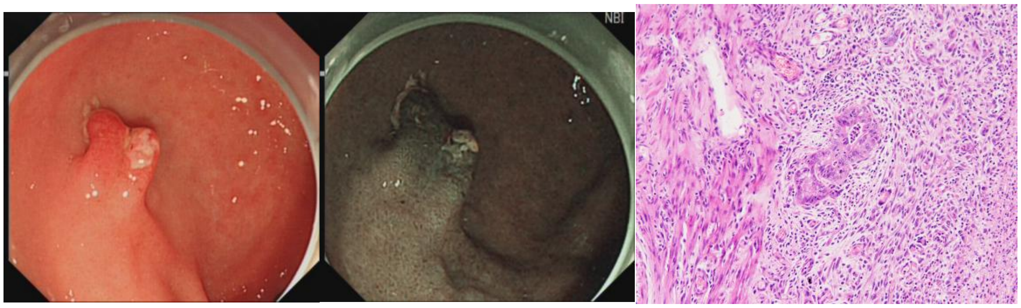

Case 9, male, 73y, 0-IIa+IIc, HGIN

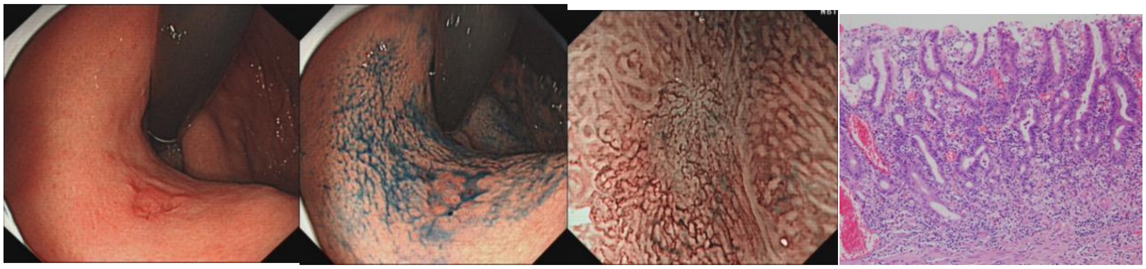

Case 10, male, 67y, 0-IIc, HGIN

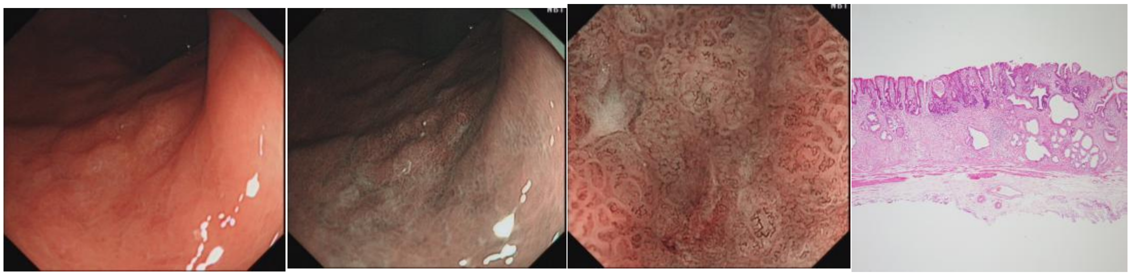

Case 11, male, 53y, 0-IIc, Tub1, 2

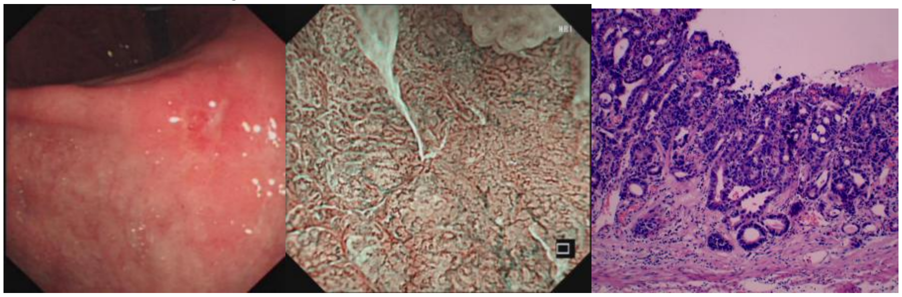

Case 12, female, 73y, 0-IIb, HGIN

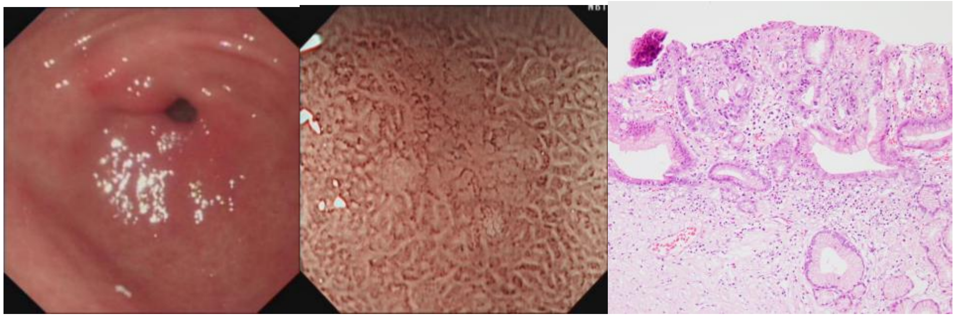

Case 13, male, 48y, 0-IIa+IIc, Tub1, 2

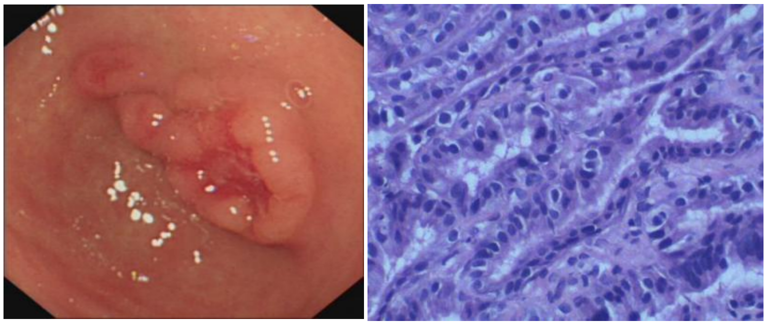

Case 14, male, 59y, 0-IIa+IIc , Tub 1

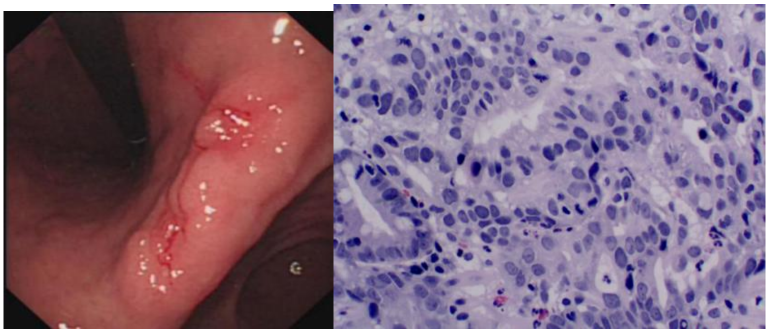

Case 15, male, 79y, 0-IIc, Tub1, 2

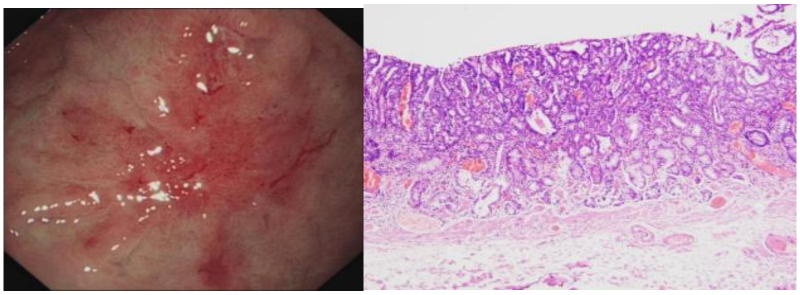

Case 16, female, 49y, 0-II a, HGIN

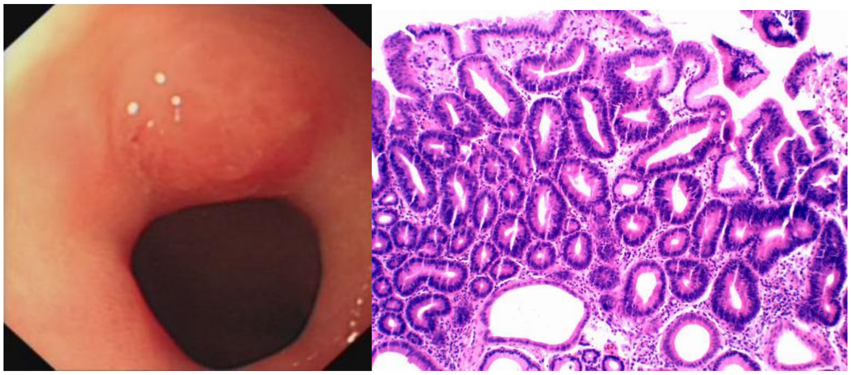

Case 17, male, 48y, 0-IIa, HGIN

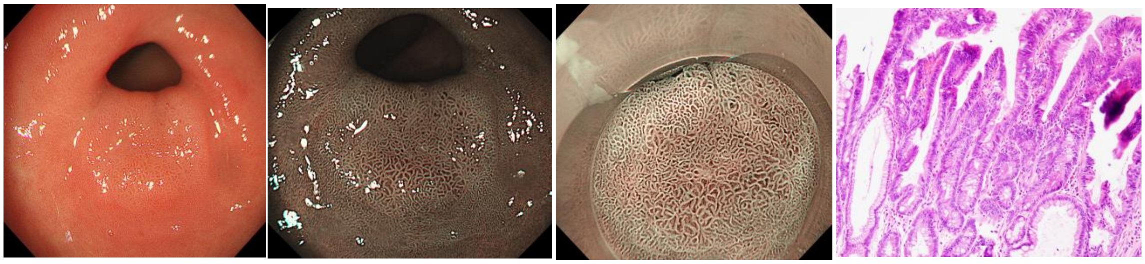

Case 18, male, 55y, 0-IIa+IIc, HGIN

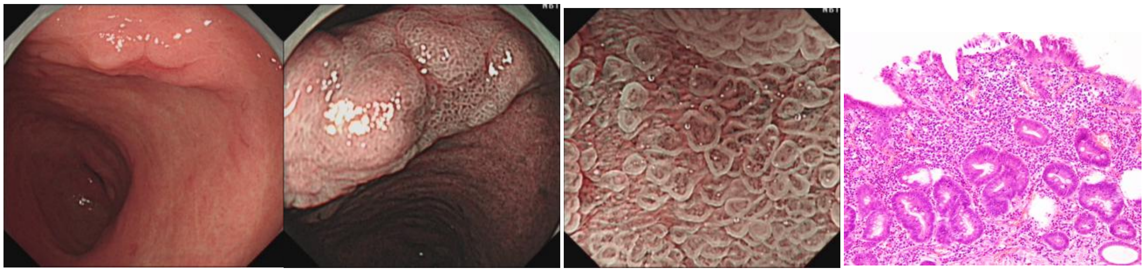

Case 19, male, 53y, 0-IIa, HGIN

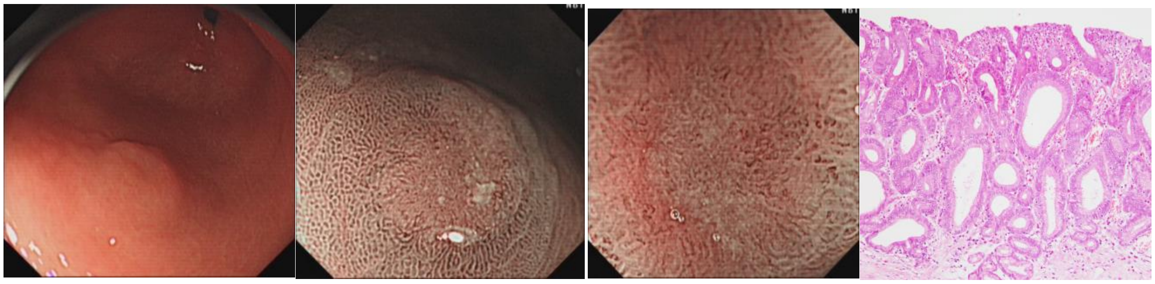

Case 20, male, 62y, 0-IIa+IIc, HGIN

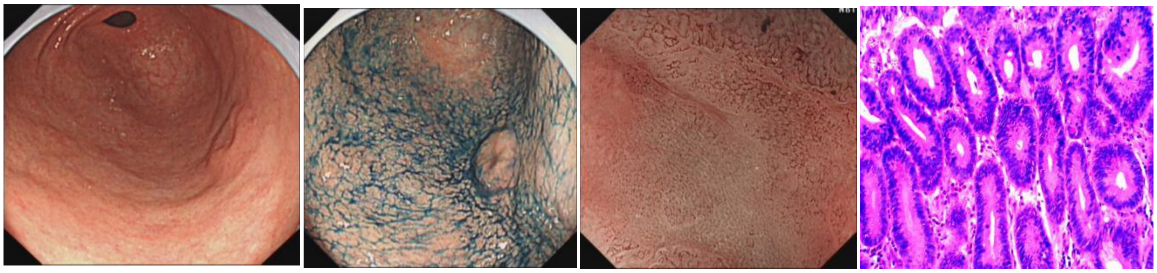

Case 21, male, 79y, 0-IIc ,HGIN

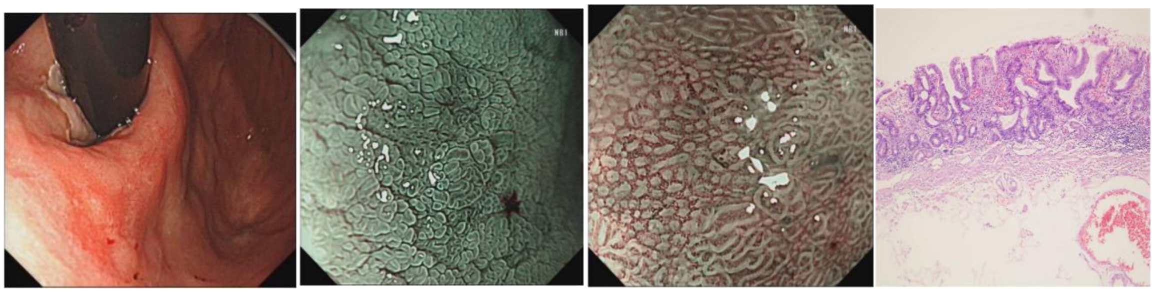

Case 22, male, 62y, 0-IIc, HGIN

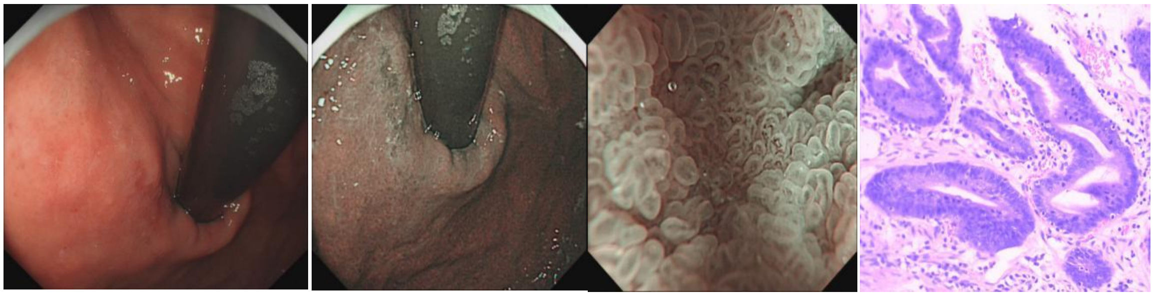

Case 23, male, 52y, 0-IIc, HGIN

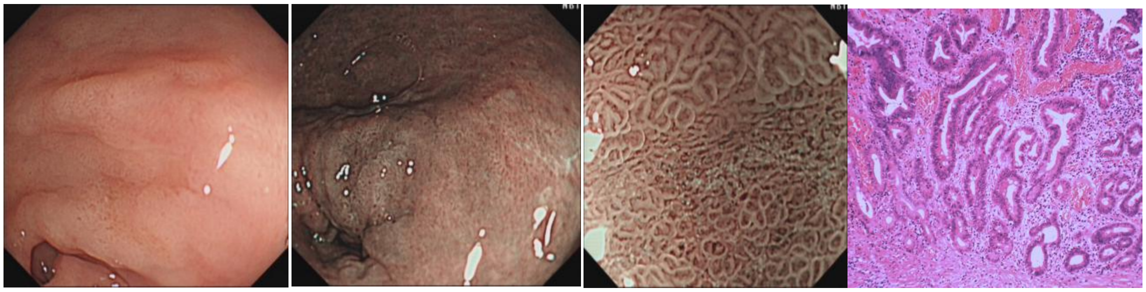

Case 24, female, 48y, 0-IIa, HGIN

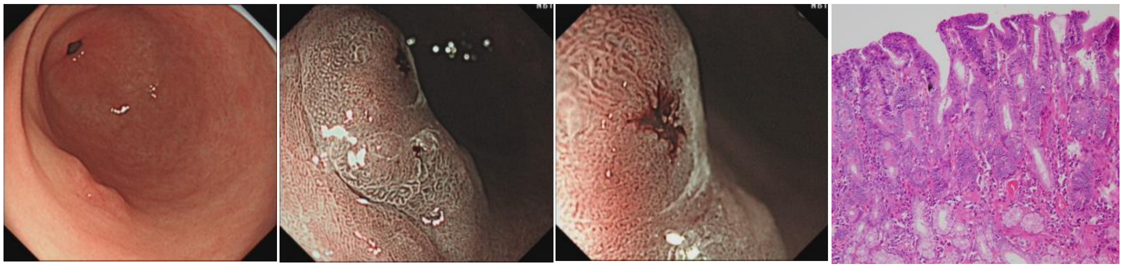

Case 25, male,52y, 0-IIa+IIc, HGIN

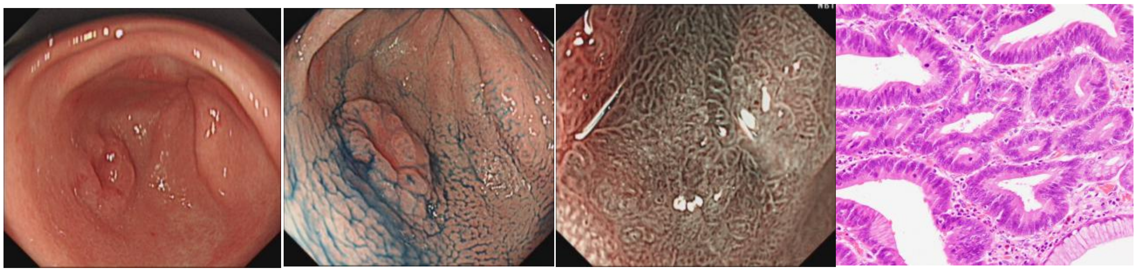

Case 26, male, 67y, 0-IIc+IIa, HGIN

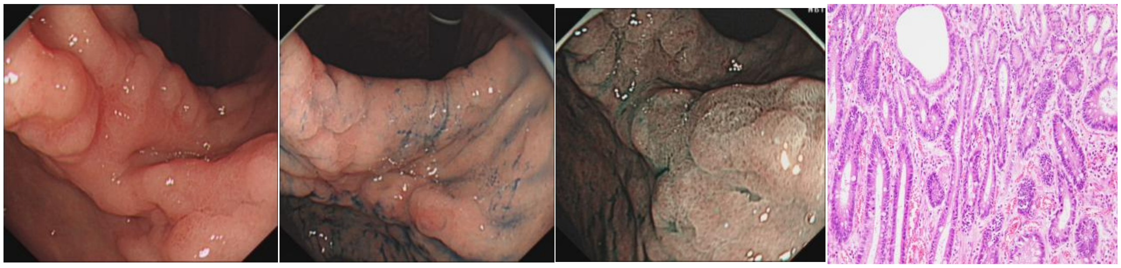

Case 27, male, 61y, 0-IIc, HGIN

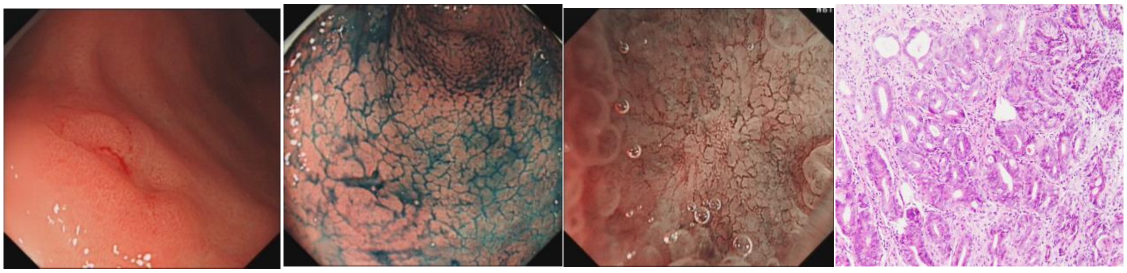

Case 28, female, 61y, 0-II a, HGIN

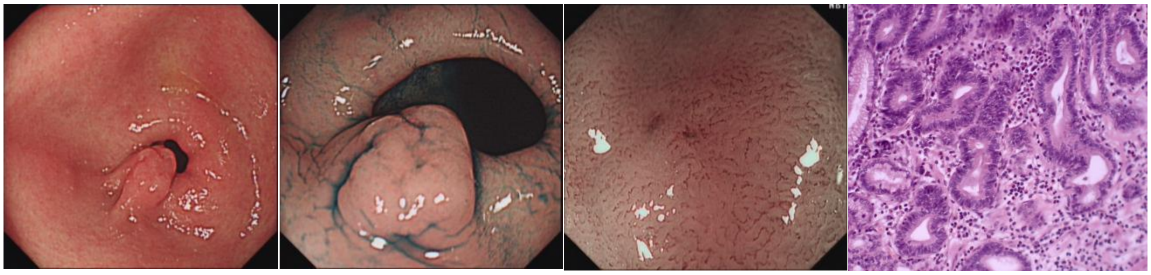

Case 29, male, 43y, 0-IIa+IIc, HGIN

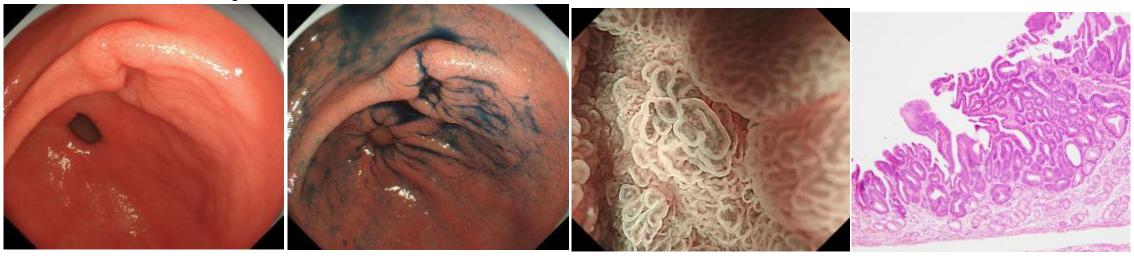

Case 30, female, 47y, 0-II a +IIc, HGIN

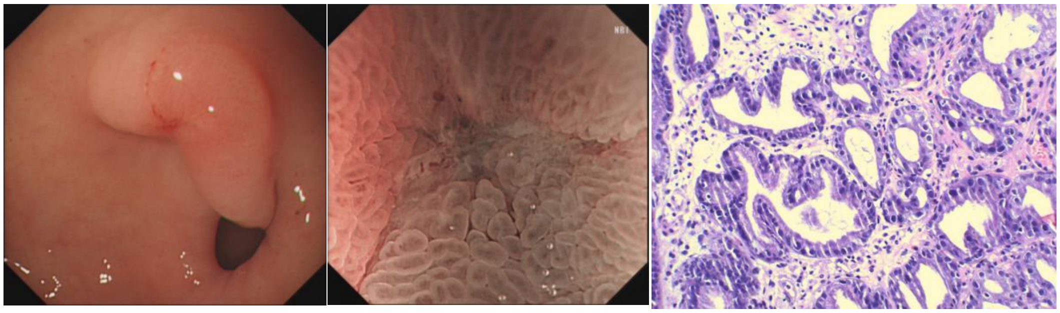

Case 31, male, 42y, 0-II a +IIc, HGIN

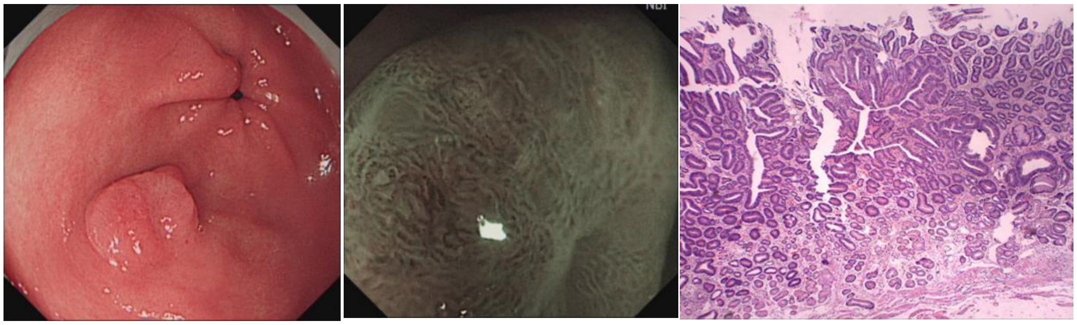

Case 32 ,male, 46y, 0-IIc, Tub 1

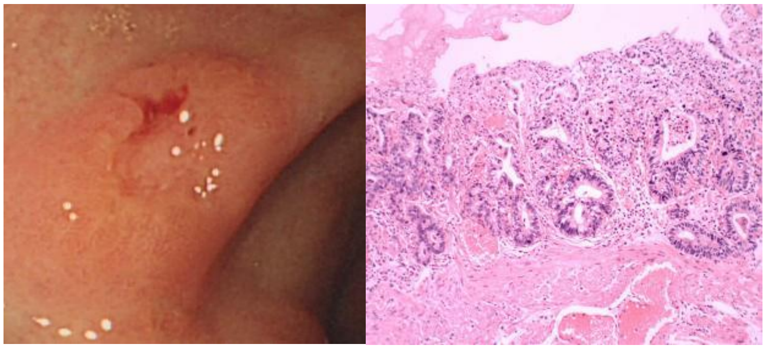

Case 33, male, 71y, 0-II a +IIc, HGIN

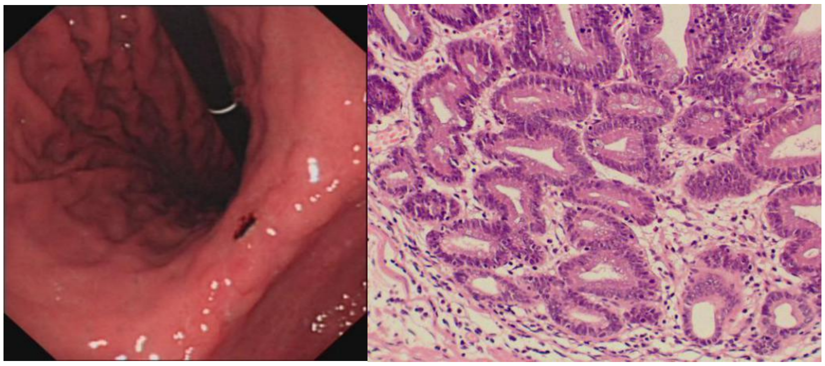

Case 34, female, 51y, 0-IIa, HGIN

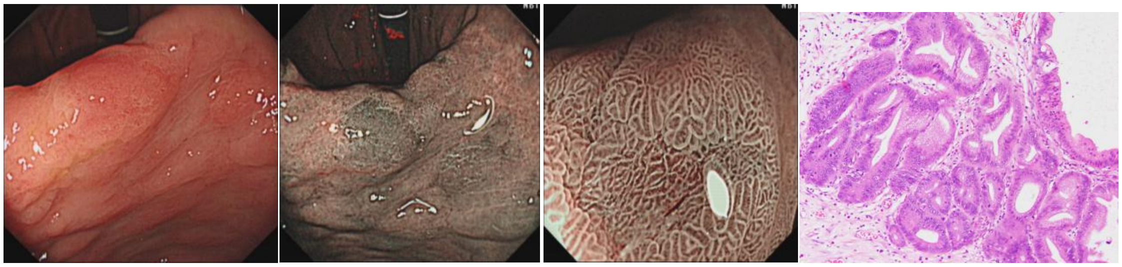

Case 35, female, 66y, 0-II a, HGIN

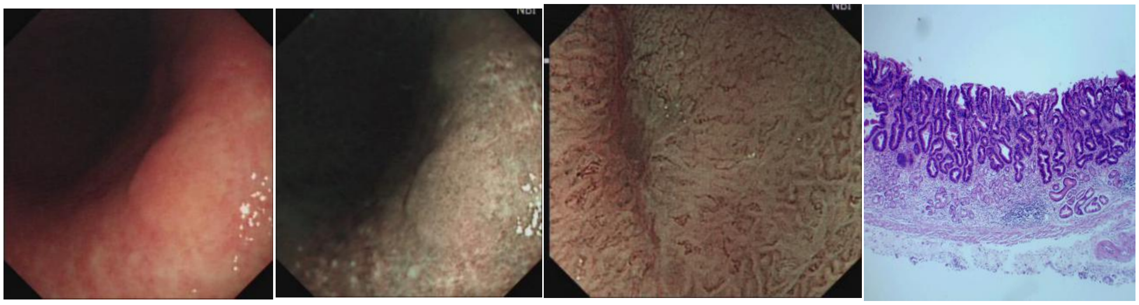

Case 36, male, 69y, 0-III, Tub 1

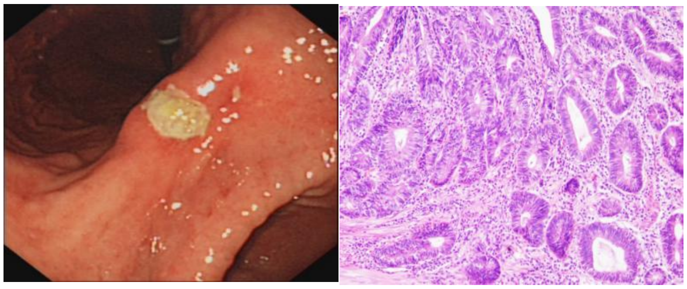

Case 37, female, 49y, 0-III, Tub1, 2

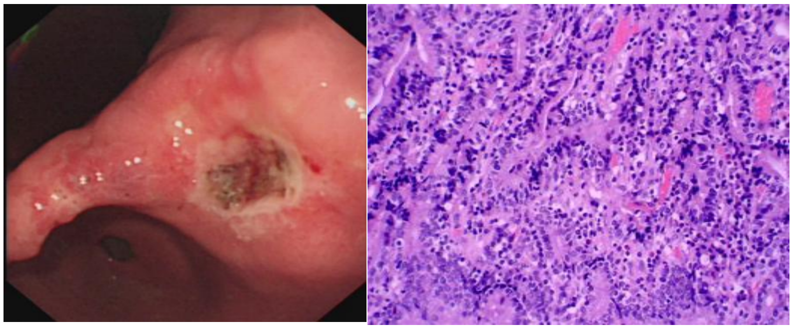

Case 38, male, 46y, 0-III, Tub 1

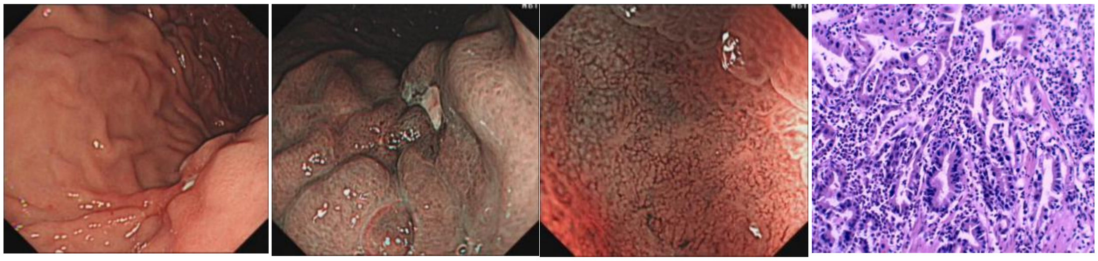

Case 39, male, 45y, 0-III, Tub1, 2

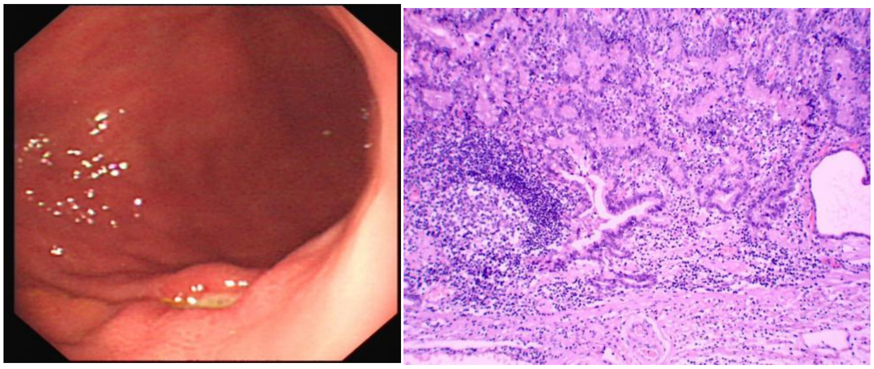

Notes: EGC, early gastric cancer; HGIN**,** high-grade intraepithelial neoplasias; MDT, multi-disciplinary team; Por 1, poorly-differentiated adenocarcinoma; Sig, signet-ring cell carcinoma; Tub1, well-differentiated adenocarcinoma; Tub 2, moderately-differentiated adenocarcinoma.
